# State Convention of Veterinary Surgeons in Chicago

**Published:** 1883-07

**Authors:** 


### STATE CONVENTION OF VETERINARY SURGEONS IN CHICAGO.

We noticed in the May number of the *United States Veterinary Journal* a call for a State convention of veterinary surgeons at Chicago, in order to adopt measures calculated to improve and protect the welfare of the profession. Prominent among the signers we notice the name of our friend, Dr. W. Sheppard, of Ottawa, a gentlemen of acknowledged ability and very enthusiastic in all that appertains to veterinary science. The Doctor was elected First Vice-President. He is a very active worker, and gave ample evidence of it, not only in directing and perfecting the organization, but in reading an essay on lameness, in order to keep the ball rolling. We congratulate the veterinary profession of Illinois for the step taken in advancing the interest and welfare of veterinary science, and in its choice of able workers. May the good work extend.—*Spirit of the Times.*

